# Supplementary material for: Formalising recall by genotype as an efficient approach to detailed phenotyping and causal inference
Source: Nat Commun. 2018 Feb 19;9:711. doi: 10.1038/s41467-018-03109-y (PMC5818506; doi:10.1038/s41467-018-03109-y)
Supplement: Supplementary file 1 — Supplementary Information [file 41467_2018_3109_MOESM1_ESM.docx]

**Supplementary Information for**

**­­­**

**Formalising recall by genotype as an efficient approach to detailed phenotyping and causal inference**

**Corbin *et al.***

# Supplementary Note 1

# Recall-by-Genotype Study Planner: *Methods for power calculation*

## RbG^sv^ – Single variant analysis

Since a ‘RbG^sv^ study’ design involves the recruitment of specific genotype groups (either major and minor homozygotes or major homozygotes and heterozygotes) resulting in two groups (independent of allele frequency), the power calculation is for an equal variance two-sample two-tailed t-test. The equation used was taken from the ‘pwr’ library^S1^ in R^S2^ and is adapted from Cohen^S3^. The non-centrality parameter (NCP, λ) is defined as:

$$\lambda=\sqrt{\left( N/2 \right)/2}*d$$

where *N* is the number of individuals in the study (total sample size) and *d* is the standardized effect size. Where the comparison is between minor and major homozygotes, the effect size will be twice the per allele effect at the target locus. Where the comparison is between major homozygotes and heterozygotes, the effect size will be equal to the per allele effect at the target locus.

For a ‘random recall study’ design, where participants are recalled randomly from the population, it is assumed that the sample will contain all three genotypic groups at frequencies determined by the user specified MAF and assuming Hardy-Weinberg equilibrium (HWE). The test of association will therefore manifest as a standard genetic association test. Power is derived from the NCP of a χ^2^-test of association (Sham & Purcell 2014)^S4^ defined as:

$$\lambda= N \times\frac{\beta^{2} Var(X)}{Residual variance Y}$$

where Y is the trait; X is the allele count at a genetic locus (coded 0, 1 or 2) so that under HWE the variance of *X* (Var(*X*)) is given by 2*p*(1 – *p*), where *p* is the allele frequency at the locus; and *β* is the regression coefficient of *Y* on *X*. For a minor-effect locus, the residual variance of *Y* is not much smaller than the total variance of *Y* ($\sigma_{Y}^{2}$), so that the NCP is given by the proportion of trait variance explained by the genetic variant multiplied by the sample size (*N*). Therefore,

$$\lambda= N \times\frac{\beta^{2} \times2p(1-p)}{\sigma_{Y}^{2}}$$

This group represents the comparator, i.e., the power to undertake a nested study of size *N* with no effort to balance genotypic groups through recall.

## RbG^mv^ – Multiple variant analysis

There are two options for estimating the power of your 'RbG^mv^ study'. The first uses simulation and the second, uses an analytical approach. Results from the two approaches are expected to be similar but may diverge where the assumptions underlying one or other of the approaches are not met, for example, when the number of SNPs in the GRS variant list is small. When there are a large number of SNPs in the GRS, using the analytical approach may be more efficient. The power calculations performed assume the assumptions of Mendelian randomization hold, specifically that the GRS only affects the outcome via the exposure and that there is no GRS-outcome confounding. We recommend only using robustly associated genetic variants in the creation of the GRS for recall studies. Including a large number of weakly associated variants in the GRS may lead to confounding. It is also assumed that both the exposure and outcome phenotypes are quantitative traits.

(A) Using simulation

If the ‘Using simulation’ option is chosen, power predictions for the 'RbG^mv^ study' are determined empirically based on simulated data. Pseudo-individuals from a population cohort (of the size specified by the user) are assigned genotypes at each of the SNPs listed in the GRS variant file according to the effect allele frequency at that SNP. A GRS is generated for each individual either by simply summing the number of risk alleles (unweighted method) or by multiplying the number of risk alleles by their corresponding weight and summing across all SNPs (weighted method).

Exposure phenotypes are simulated by adding a random (normally distributed) error term, scaled according to the user-entered R^2^ between the GRS and exposure phenotype. Outcome phenotypes are simulated by adding a random (normally distributed) error term scaled according to the user-entered R^2^ between the exposure and outcome phenotypes to the previously simulated exposure phenotypes. This procedure is repeated to generate 25 pseudo-populations.

Assuming the random recruitment of individuals from the tails of the GRS distribution (percentile (%) as specified by user) 1,000 pseudo-datasets are created (per simulated population) by randomly selecting n/2 individuals (where ‘n’ is the proposed sample size) from each of the two tails (assuming equal recruitment). This simulated data is used firstly to evaluate the power of a 'RbG^mv^ study' design to detect a difference in mean exposure phenotype across the strata generated by selecting individuals from the tails of the simulated GRS distribution. Assuming the variance in exposure explained by the GRS is small, we can assume that Y is approximately normal in both groups and therefore an equal variance two-sample two-sided t-test is used to test for a difference in mean exposure phenotype across the two recall groups. The power is estimated as the proportion of test results less than the user specified alpha level across all simulated populations and datasets. This procedure is then repeated to evaluate the power of the same 'RbG^mv^ study' design to detect a difference in mean outcome phenotype across the two recall groups.

In addition, we consider the relative power if the same study was performed in either a randomly recruited sample of the same size as the ‘RbG^mv^ study’ (‘random recall study’) or a (genotyped) population cohort of the size specified for recruitment (‘total cohort study’). In the case of the ‘random recall study’, 1,000 pseudo-datasets are created (per simulated population) by randomly selecting ‘n’ individuals (where ‘n’ is the proposed sample size) from across the entire GRS distribution. In the case of the ‘total cohort study’, all individuals are included in the analysis. A linear regression model is then used to test for a relationship between the GRS and the outcome phenotype. The power is estimated as the proportion of test results less than the user specified alpha level across all simulated populations and datasets.

(B) Using an analytical approach

If the ‘Using an analytical approach’ option is chosen, power predictions for the 'RbG^mv^ study' are determined analytically. Assuming the GRS is standard normal, let the variance in exposure explained by the GRS be $R_{XG}^{2}$. Assuming the exposure is standard normal then the exposure model is:

$$X= R_{XG}G+ \in_{X}$$

Assume the outcome is standard normal then the outcome model is:

$$Y= {R_{YX}X+ \in_{Y} = R_{YX}R}_{XG}G+ R_{YX}\in_{X}+ \in_{Y}$$

If the top and bottom 100*q*% (percentile) of the GRS is selected, let $t= \Phi^{-1}(q)$. From properties of the truncated normal distribution, the mean GRS in the top 100*q*% is $\frac{\phi(t)}{\Phi(t)}$ and the variance is $1-\frac{t\phi\left( t \right)}{\Phi\left( t \right)}- \left[ \frac{\phi(t)}{\Phi(t)} \right]^{2}$. So the mean Y of this group is $R_{YX}R_{XG}\frac{\phi(t)}{\Phi(t)}$ and the variance is $R_{YX}^{2}R_{XG}^{2}\left( 1-\frac{t\phi\left( t \right)}{\Phi\left( t \right)} - \left[ \frac{\phi(t)}{\Phi(t)} \right]^{2} \right)+R_{YX}^{2}\left( 1-R_{XG}^{2} \right)+ \left( 1-R_{YX}^{2} \right)$. Similarly, the mean of the GRS in the bottom 100*q*% is $\frac{-\phi(t)}{\Phi(t)}$ and the variance is $1-\frac{t\phi\left( t \right)}{\Phi\left( t \right)}- \left[ \frac{\phi(t)}{\Phi(t)} \right]^{2}$. So the mean Y of this group is ${-R}_{YX}R_{XG}\frac{\phi(t)}{\Phi(t)}$ and the variance is also $R_{YX}^{2}R_{XG}^{2}\left( 1-\frac{t\phi\left( t \right)}{\Phi\left( t \right)} - \left[ \frac{\phi(t)}{\Phi(t)} \right]^{2} \right)+R_{YX}^{2}\left( 1-R_{XG}^{2} \right)+ \left( 1-R_{YX}^{2} \right)$. If $R_{XG}^{2}$ is small we can assume that Y is approximately normal in both groups, and apply the equal variances two-sample two-sided t-test for a difference in means, this difference being $2R_{YX}R_{XG}\frac{\phi(t)}{\Phi(t)}$ .

In the case of the ‘random recall study’ and the ‘total cohort study’, power is derived analytically using the NCP of the χ^2^-test of association between the outcome phenotype and the GRS. The equation used assumes an additive genetic model for a quantitative trait^S5,S6^ and is presented in Palla and Dudbridge (2015)^S7^ with the NCP defined as:

$$\lambda= \frac{N* R^{2}}{(1-R^{2})}$$

where *N* is the number of individuals (in the case of the ‘random recall study’ this is the proposed sample size of the 'RbG^mv^ study' and in the case of the ‘total cohort study’ this is the size of the total (genotyped) population cohort) and *R^2^* is the coefficient of determination between the GRS and either the exposure phenotype or the outcome phenotype; in the case of the latter, the *R^2^* between the GRS and the outcome phenotype is calculated as the product of the *R^2^* between the GRS and the exposure phenotype and the *R^2^* between the exposure phenotype and the outcome phenotype (both provided by the user).

**Supplementary Note 2**

**Data description for Figure 2**

The data used to produce Figure 2 in the main text was sourced from mothers recruited by the Avon Longitudinal Study of Parents and Children (ALSPAC). The full details about this cohort can be found below. Physical measures (body mass index (BMI) and systolic blood pressure (SBP)) were recorded at a follow-up clinic carried out approximately 16 years after the mother’s pregnancy. Information about confounding factors was taken from questionnaires completed by the mothers either during pregnancy (education, defined as the mother’s highest qualification) or when their child was aged 18 years (frequency of alcohol consumption and average take-home household income each month). Pre-existing genetic data (for details of the SNP genotyping, imputation, processing and quality control procedures carried out in ALSPAC see below) was used to generate the genetic risk score (GRS) for BMI based on 97 SNPs^S8^. Data analysis was conducted in STATA^S9^ v14.2.

*ALSPAC: Description of study numbers*

ALSPAC recruited 14,541 pregnant women resident in Avon, UK with expected dates of delivery 1st April 1991 to 31st December 1992. 14,541 is the *initial* number of pregnancies for which the mother enrolled in the ALSPAC study and had either returned at least one questionnaire or attended a “Children in Focus” clinic by 19/07/99. Of these *initial* pregnancies, there was a total of 14,676 fetuses, resulting in 14,062 live births and 13,988 children who were alive at 1 year of age.

When the oldest children were approximately 7 years of age, an attempt was made to bolster the initial sample with eligible cases who had failed to join the study originally. As a result, when considering variables collected from the age of seven onwards (and potentially abstracted from obstetric notes) there are data available for more than the 14,541 pregnancies mentioned above.

The number of **new pregnancies** not in the initial sample (known as Phase I enrolment) that are currently represented on the built files and reflecting enrolment status at the age of 18 is 706 (452 and 254 recruited during Phases II and III respectively), resulting in an additional 713 children being enrolled. The phases of enrolment are described in more detail in the cohort profile paper which should be used for referencing purposes: <http://ije.oxfordjournals.org/content/early/2012/04/14/ije.dys064.full.pdf>.

The total sample size for analyses using any data collected after the age of seven is therefore 15,247 pregnancies, resulting in 15,458 fetuses. Of this **total sample** of 15,458 fetuses, 14,775 were **live births** and 14,701 were **alive at 1 year of age**.

A 10% sample of the ALSPAC cohort, known as the **Children in Focus (CiF) group**, attended clinics at the University of Bristol at various time intervals between 4 to 61 months of age. The CiF group were chosen at random from the last 6 months of ALSPAC births (1432 families attended at least one clinic). Excluded were those mothers who had moved out of the area or were lost to follow-up and those partaking in another study of infant development in Avon.

*ALSPAC: Genotyping description*

ALSPAC children were genotyped using the Illumina HumanHap550 quad chip genotyping platforms by 23andme subcontracting the Wellcome Trust Sanger Institute, Cambridge, UK and the Laboratory Corporation of America, Burlington, NC, US. The resulting raw genome-wide data were subjected to standard quality control methods. Individuals were excluded on the basis of gender mismatches; minimal or excessive heterozygosity; disproportionate levels of individual missingness (>3%) and insufficient sample replication (IBD < 0.8). Population stratification was assessed by multidimensional scaling analysis and compared with Hapmap II (release 22) European descent (CEU), Han Chinese, Japanese and Yoruba reference populations; all individuals with non-European ancestry were removed. SNPs with a minor allele frequency of < 1%, a call rate of < 95% or evidence for violations of Hardy-Weinberg equilibrium (P < 5E-7) were removed. Cryptic relatedness was measured as proportion of identity by descent (IBD > 0.1). Related subjects that passed all other quality control thresholds were retained during subsequent phasing and imputation. 9,115 subjects and 500,527 SNPs passed these quality control filters.

ALSPAC mothers were genotyped using the Illumina human660W-quad array at Centre National de Génotypage (CNG) and genotypes were called with Illumina GenomeStudio. PLINK^S10^ (v1.07) was used to carry out quality control measures on an initial set of 10,015 subjects and 557,124 directly genotyped SNPs. SNPs were removed if they displayed more than 5% missingness or a Hardy-Weinberg equilibrium P-value of < 1E-6. Additionally SNPs with a minor allele frequency of less than 1% were removed. Samples were excluded if they displayed more than 5% missingness, had indeterminate X chromosome heterozygosity or extreme autosomal heterozygosity. Samples showing evidence of population stratification were identified by multidimensional scaling of genome-wide identity by state pairwise distances using the four HapMap populations as a reference and then excluded. Cryptic relatedness was assessed using a IBD estimate of more than 0.125 which is expected to correspond to roughly 12.5% alleles shared IBD or a relatedness at the first cousin level. Related subjects that passed all other quality control thresholds were retained during subsequent phasing and imputation. 9,048 subjects and 526,688 SNPs passed these quality control filters.

*ALSPAC: Imputation description*

477,482 SNP genotypes in common between the sample of mothers and sample of children were combined. SNPs with genotype missingness above 1% due to poor quality were removed (11,396 SNPs removed). 321 subjects were removed due to potential ID mismatches. This resulted in a dataset of 17,842 subjects containing 6,305 duos and 465,740 SNPs (112 were removed during liftover and 234 were out of HWE after combination). Haplotypes were estimated using ShapeIT (v2.r644) which utilises relatedness during phasing. A phased version of the 1000 genomes reference panel (Phase 1, Version 3) was obtained from the Impute2 reference data repository (phased using ShapeIt v2.r644, haplotype release date Dec 2013). Imputation of the target data was performed using IMPUTE^S11, S12^ V2.2.2 against the reference panel (all polymorphic SNPs excluding singletons), using all 2,186 reference haplotypes (including non-Europeans). This gave 17,842 mothers and children eligible for study with available genotype data. Subsequent consent withdrawals have left 17,825 individuals for study.

**Supplementary Table 1.** UK patient and population-based studies available for RbG studies (extended version of Table 1).

| **Study** | **In NIHR Bio-resource** | **Genetic data type** | **MAF range** | **Sample size** | **Resource summary publication or website** | **PI / Contact point** | **Local phenotypic expertise** | **Local consent policy and requirements for RbG** | **Samples available** | **Patient group/ population sample** |
| --- | --- | --- | --- | --- | --- | --- | --- | --- | --- | --- |
| The Avon Longitudinal Study of Parents and Children  (ALSPAC) | No | GWAS  (imputed up to 1000g/1000g-UK10K/HRC & Low read depth NGS (n=2,000) | ~0.005- | ~9,000 (mother child duos) & ~2,000 trios. Smaller number of children of index participants (third gen) | www.bris.ac.uk/alspac | Nicholas J. Timpson  (contact: Nicholas J. Timpson, n.j.timpson@bris.ac.uk) | Lifecourse epidemiology  -birth cohort ("complete" phenotyping) | LREC  - RbG studies have been undertaken.  (Non-disclosure) | Blood spot (multiple ages), serum (multiple ages), LCL (on nearly all), whole blood on some and third gen gut samples;  Others charted at web-repository. | Population-based cohort |
| East London Genes & Health  (ELGH) | No | Exome sequencing on 4,561  (at Nov 2017)  Applying for funding for SNP array genotyping. | Includes rare variants in homozygous (autozygous) state due to parental  relatedness | 26,476  (at Nov 2017, actively recruiting, total sample size 100k) | www.genesandhealth.org | David A. van Heel,  Richard Trembath  (contact: David A. van Heel, d.vanheel@qmul.ac.uk) | Human knockouts, primary care e-health records, diabetes and cardiovascular | Application/ Approval process; Recall up to 4 times/year per volunteer | Oragene saliva and DNA  (Near future: Stored blood on a subset. Lipid, HbA1C etc. data via EHR. Subset also undergoing retinal photo, iDXA, liver Fibroscan, bioimpedance, BMI/waist circumference). | Population-based cohort  (Bangladeshi and Pakistani ethnicity, age>16) |
| EXtended Cohort  for E-health, Enviroment  and DNA  (EXCEED) | No | GWAS data (Affymetrix Axiom UK Biobank array) for first 6,000 with imputation to HRC | Includes “exome array” style rare variants and grid of variants optimised for imputation of MAF 1-5% | Over 9,300 recruits to date; recruitment planned to continue to 10,000 | http://www.leicsrespiratorybru.nihr.ac.uk/our-research/our-research-studies/exceed/ | Martin D. Tobin  (contact: exceed@le.ac.uk) | Cardiovascular, respiratory, renal, metabolic, infectious disease and cancer | Full consent from participants for RbG is in place; Consent includes option for patients to decline feedback of genetic information. | Stored DNA  from saliva  (in UK Biocentre) | Population-based cohort (aged 30-69) |
| Exeter 10,000 (EXTEND) | No | Illumina HumanCore, exome chip  and Global screening arrays | 0.001-0.5 | 10,000 | http://exeter.crf.nihr.ac.uk/content/about-exeter-crf | Tim Frayling,  Andrew Hattersley  (Contact: Tim Frayling, T.M.Frayling@exeter.ac.uk) | Type 2 diabetes, ischaemic heart disease, vascular function and healthy ageing | Dynamic consent by proxy.  (See DOI 10.1186/s40900-015-0002-y  Jenner et al 2015.) | Frozen plasma, serum and DNA | Population-based sample (based in Exeter; enriched for patients with diabetes; aged>18) |
| Genetics of Diabetes and Audit Research Tayside Study (GoDARTS) | No | GWAS/exome array/metabochip/  Imputed to HRC 65K | all | 9,439 cases and 8,187 controls | http://diabetesgenetics.  dundee.ac.uk | Colin N.A. Palmer, Ewan R. Pearson (Contact: Colin N.A. Palmer, c.n.a.palmer@dundee. ac.uk) | Complete EMR linkage, type 2 diabetes, heart disease, asthma and cancer | Applications through GODARTS Access committee;  Full consent  for RbG. | Frozen serum, blood and DNA | Case-control cohort |
| INTERVAL | Yes  (ongoing) | GWAS data (Affymetrix Axiom UK Biobank array) imputed to 1000G+UK10K, 50x WES in 4,500 participants, 15x WGS (ongoing) | all | 50,000 | http://www.intervalstudy.  org.uk/ | John Danesh  (Contact: helpdesk@intervalstudy.org) | >6,000 molecular phenotypes, including serum NMR metabolomics, plasma MS lipidomics and metabolomics, plasma proteomics, Sysmex FBC, hepcidin and others | LREC/HRA for RbG studies, recruitment is through the NIHR BioResource for Translational Research in Common and Rare Diseases | DNA, serum, plasma, buffy coat, RNA (stabilized) | Population-based sample of healthy blood donors |
| National Centre for Mental Health | No | Genotypic / GWAS data on Illumina psych/DPMCN chips | 0.005 | Over 10,000 | http://ncmh.info/biobank/ | Ian Jones  (Contact: info@ncmh.info) | Mental health conditions. | Full consent taken;  HTA-approved application for access system in place | DNA, blood  and saliva. | Population-based cohort (variety of mental health conditions; all ages; primarily Wales-based) |
| The Oxford Biobank | No | Illumina Exome chip (n=5,500), Affymetrix Axiom (UK Biobank Array, n=7,500) | ~0.005- | 7,900 | www.oxfordbiobank.org.uk | Fredrik Karpe, Matt Neville  (contact: Matt Neville, Matthew.Neville@ocdem.ox.ac.uk or obb@ocdem.ox.ac.uk) | Metabolic and anthropometric, obesity | Consent for recall in general, but new studies may require new approvals depending on research question and sample requirement; Existing consent for single blood sample. | Frozen plasma (Heparin and EDTA) and serum;  DNA ready for  on-demand  high-throughput genotyping; Extensive phenotypes available. | Random, population-based sample of healthy 30-50y men  and women (Oxfordshire) |
| Scottish Health Research Register (SHARE) | No | Limited GWAS available. | all | 50,000 samples obtained.  155,000 consented for spare blood interception | http://www.registerforshare.  org/index.php | Colin Palmer  (Contact: Colin Palmer, c.n.a.palmer@dundee. ac.uk) | Complete EMR linkage. Type 2 diabetes, heart disease, asthma and cancer. Mobile App Patient Reported Outcomes. | Applications through GoSHARE Access committee; Consent for  Re-contact by Genotype;  Up to 4 contacts per year. | Frozen serum, blood, DNA. Automated longitudinal sampling  available | Population-based cohort |
| Generation Scotland: Scottish Family Health Study  (GS:SFHS) | No | Illumina Omni express+Exome Imputed to HRC | all | 20,032 | http://www.ed.ac.uk/generation-scotland | Caroline Hayward/ Caroline.Hayward@igmm.ed.ac.uk | Complete EHR linkage, urinary traits & kidney disease, eye phenotypes, family based data analysis | Applications through GS Access Committee. Full consent for RbG. | Frozen serum & urine, DNA | Family-based population cohort |

1000g, 1000 genomes; BRC, Biomedical research centre; EDTA, ethylenediaminetetraacetic acid; EHR, electronic health record; EMR, electronic medical records; FBC, full blood count; GWAS, Genome-wide association study; HRA, health research authority; HRC, Haplotype reference consortium; HTA, human tissue authority; LCL, lymphoblastoid cell lines; LREC, local research ethics committees; MS, mass spectrometry; NGS, next generation sequencing; NIHR, National Institute for Health Research; NMR, nuclear magnetic resonance; RbG, Recall-by-Genotype

**Supplemental References:**

S1 - Champely S. (2015). pwr: Basic Functions for Power Analysis. R package version 1.1-3. https://CRAN.R-project.org/package=pwr

S2 - R Core Team (2016). R: A language and environment for statistical computing. R Foundation for Statistical Computing, Vienna, Austria. URL https://www.R-project.org/.

S3 - Cohen, J. (1988). Statistical power analysis for the behavioral sciences (2nd ed.). Hillsdale, NJ: Lawrence Erlbaum.

S4 - Sham P.C., Purcell S.M. (2014). Statistical power and significance testing in large-scale genetic studies. *Nature Reviews Genetics* 15, 335–346

S5 - Daetwyler H. D., Villanueva B., Woolliams J.A. (2008). Accuracy of predicting the genetic risk of disease using a genome-wide approach. *PLoS ONE* 3, e3395.

S6 - Dudbridge F. (2013) Power and predictive accuracy of polygenic risk scores. *PLoS Genetics* 9, e1003348.

S7 - Palla L. and Dudbridge F. (2015). A fast method that uses polygenic scores to estimate the variance explained by genome-wide marker panels and the proportion of variants affecting a trait. *The American Journal of Human Genetics* 97, 250-259.

S8 - Locke A.E., Kahali B., Berndt S.I., Justice A.E., Pers T.H., Day F.R., Powell C., Vedantam S., Buchkovich M.L., Yang J., *et al.* (2015) Genetic studies of body mass index yield new insights for obesity biology. *Nature* 518, 197-206.

S9 - StataCorp. (2015) *Stata Statistical Software: Release 14*. College Station, TX: StataCorp LP.

S10 - Purcell S., Neale B., Todd-Brown K., Thomas L., Ferreira M.A., Bender D., Maller J., Sklar P., de Bakker P.I., Daly M.J., *et al.* (2007) PLINK: A Tool Set for Whole-Genome Association and Population-Based Linkage Analyses. *The American Journal of Human Genetics* 81, 559-575.

S11 - Howie B.N., Donnelly P. and Marchini J. (2009) A flexible and accurate genotype imputation method for the next generation of genome-wide association studies. *PLoS Genetics* 5, e1000529.

S12 - Howie B.N., Marchini J. and Stephens M. (2011) Genotype imputation with thousands of genomes. *G3: Genes, Genomes, Genetics* 1, 457-470.
